# Methods to Study Intracellular Movement and Localization of the Nucleotide Excision Repair Proteins at the DNA Lesions in Mammalian Cells

**DOI:** 10.3389/fcell.2020.590242

**Published:** 2020-11-17

**Authors:** Mihaela Robu, Rashmi G. Shah, Girish M. Shah

**Affiliations:** CHU de Québec Université Laval Research Centre (site CHUL), Laboratory for Skin Cancer Research and Axe Neuroscience, Québec, QC, Canada

**Keywords:** nucleotide excision repair (NER), NER proteins, poly(ADPR-ribose) polymerase-1 (PARP1), localization at DNA damage, local irradiation, *in situ* extraction, sub-cellular fractionation, intracellular movement

## Abstract

Nucleotide excision repair (NER) is the most versatile DNA repair pathway that removes a wide variety of DNA lesions caused by different types of physical and chemical agents, such as ultraviolet radiation (UV), environmental carcinogen benzo[a]pyrene and anti-cancer drug carboplatin. The mammalian NER utilizes more than 30 proteins, in a multi-step process that begins with the lesion recognition within seconds of DNA damage to completion of repair after few hours to several days. The core proteins and their biochemical reactions are known from in vitro DNA repair assays using purified proteins, but challenge was to understand the dynamics of their rapid recruitment and departure from the lesion site and their coordination with other proteins and post-translational modifications to execute the sequential steps of repair. Here, we provide a brief overview of various techniques developed by different groups over last 20 years to overcome these challenges. However, more work is needed for a comprehensive knowledge of all aspects of mammalian NER. With this aim, here we provide detailed protocols of three simple yet innovative methods developed by many teams that range from local UVC irradiation to in situ extraction and sub-cellular fractionation that will permit study of endogenous as well as exogenous NER proteins in any cellular model. These methods do not require unique reagents or specialized instruments, and will allow many more laboratories to explore this repair pathway in different models. These techniques would reveal intracellular movement of these proteins to the DNA lesion site, their interactions with other proteins during repair and the effect of post-translational modifications on their functions. We also describe how these methods led us to identify hitherto unexpected role of poly(ADP-ribose) polymerase-1 (PARP1) in NER. Collectively these three simple techniques can provide an initial assessment of the functions of known and unknown proteins in the core or auxiliary events associated with mammalian NER. The results from these techniques could serve as a solid foundation and a justification for more detailed studies in NER using specialized reagents and more sophisticated tools. They can also be suitably modified to study other cellular processes beyond DNA repair.

## Introduction

The nucleotide excision repair (NER) is the most versatile DNA repair pathway that eliminates a wide variety of DNA lesions caused by different types of physical and chemical agents, such as ultraviolet radiation (UV), environmental carcinogen benzo[a]pyrene and anti-cancer drug carboplatin. It is the only repair pathway in mammalian cell that removes UV-induced DNA damage, such as cyclobutane pyrimidine dimers (CPD) and pyrimidine (6-4) pyrimidone (6-4PP) photoproducts (Scharer, 2013). The photosensitivity and susceptibility to develop sunlight-induced skin cancers in the individuals carrying mutations in NER genes, such as Xeroderma pigmentosum (XP) strongly indicates the importance of NER in the repair of UV damaged DNA (Giordano et al., 2016). The NER pathway uses more than 30 proteins to recognize the lesion, remove 24-32 nucleotides from the strand containing the damage, synthesize a new strand using the undamaged strand as a template and fill the gap (Marteijn et al., 2014). NER is divided into two sub-pathways: transcription-coupled repair (TCR) and global genome repair (GGR). TCR rapidly removes lesions that efficiently block the elongating RNA polymerase II complex during transcription, while GGR occurs in the whole genome (Vermeulen and Fousteri, 2013). These two sub-pathways differ in the lesion recognitions step, and subsequently converge to complete the repair process, as briefly summarized below.

The Xeroderma pigmentosum C (XPC) protein starts the GGR sub-pathway of NER by recognizing the distortion of the DNA double helix and binding to the unpaired nucleotides facing the damaged nucleotide. Its arrival at the damage site is a prerequisite for the recruitment of downstream proteins and the repair of the lesion (Puumalainen et al., 2014). In the mammalian cells, XPC’s task of rapidly finding and localizing at the lesion site in the chromatin context is helped by UV-damaged DNA binding (UV-DDB) complex (DDB1 and DDB2) and poly(ADP-ribose) polymerase-1 (PARP1) (Pines et al., 2013). Following UV irradiation, PARP1 and DDB2 arrive rapidly and independently at the lesion, and influence each other’s functions. PARP1 stabilizes DDB2 at the lesion and DDB2 stimulates PARP1’s catalytic activity (Luijsterburg et al., 2012; Pines et al., 2012; Robu et al., 2013). PARP1 cleaves the substrate nicotinamide adenine dinucleotide (NAD^+^) and transfers the ADP-ribose moieties to poly(ADP-ribosyl)ate or PARylate itself and DDB2 as well as other acceptors proteins (Barkauskaite et al., 2015; Ray Chaudhuri and Nussenzweig, 2017). DDB2 activates the Cul4A-RBX1 ubiquitin ligase complex, containing DDB2, DDB1, Cul4A, and Rbx1 (Groisman et al., 2003) to modify histones, Cul4A and DDB2 itself. Together, these post-translational modifications (PTM) and the chromatin remodeling around the lesion facilitate recruitment and stabilization of XPC at the site. In addition, XPC forms a complex with PARP1 in the nucleoplasm prior to irradiation and is escorted rapidly by PARP1 to the lesion site after UV damage, thus improving the efficiency of initiation of GGR (Robu et al., 2017). The TCR pathway, on the other hand, is initiated when the elongating RNA polymerase II stalls at the lesion site and recruits Cockayne syndrome B (CSB) that is involved with other partners in recognition of the damage and remodeling of chromatin (Van Der Weegen et al., 2020). Once the damage is recognized, the GGR and TCR sub-pathways converge with the recruitment of the RPA, XPA, and the basal transcription factor TFIIH to verify the damage. The dual incision of damaged DNA by endonucleases XPF-ERCC1 and XPG, followed by gap filling by different polymerases (polymerses δ, ε, and κ) and DNA ligation by ligase I or XRCC1-ligase III complex complete the repair (Mullenders, 2018).

The progress in understanding mammalian NER was relatively slower than other DNA repair pathways, largely because of a conceptual and some technical hurdles. In the early stage, the specific biochemical reactions carried out by a majority of the mammalian NER proteins were identified using the in vitro DNA repair assays with purified proteins and from their equivalent proteins in bacteria and yeast (Aboussekhra et al., 1995; Sugasawa et al., 1998; Ticli and Prosperi, 2019). However, these techniques lack the spatio-temporal properties (Ticli and Prosperi, 2019). Hence, the bigger challenge was to understand the dynamics of their rapid recruitment to the lesion site and coordination of sequential steps of NER along with other proteins in mammalian cells. Based on yeast models of NER (Svejstrup et al., 1995) and some mammalian studies (He and Ingles, 1997), it was proposed that human cells carry out NER by “repairosome,” a multi-protein complex containing most of the NER proteins. Considering that mammalian NER is initiated within seconds after DNA damage and continues for several hours, this concept posed a logistical challenge of keeping many of these multi-functional NER proteins engaged in a repairosome for the entire period of repair before and after their task is required. This model also hindered the discovery of new mammalian NER proteins, if they were not previously identified as a member of the mammalian repairosome complex. Despite accumulating evidence to the contrary, as described below, the concept of repairosome carrying out mammalian NER in human cells prevailed until 2003 Friedberg (2003).

A series of innovative methods developed over the last two decades by many groups allowed a rapid gain in our understanding of mammalian NER and challenged the repairosome concept. The development of green fluorescent protein (GFP) technology and photobleaching procedures permitted the visualization and quantification of the mobility of GFP-tagged NER factors (Vermeulen, 2011). Based on the speed of the recovery of the fluorescence of GFP-XPF-ERCC1 complex in the bleached area, Houtsmuller et al. (1999) concluded that ERCC1/XPF was not part of a large NER holocomplex or “repairosome.” The same study also revealed the second hurdle that NER is not spatially constrained to sub-nuclear structures or “foci.” This was because the uniform distribution pattern of the GFP-tagged NER proteins, seen in the unirradiated nuclei remained unchanged after global UVC-irradiation. In contrast, after the global exposure of cells with ionizing irradiation, etoposide or topoisomerase inhibitors, the double strand break (DSB) repair proteins accumulate in specific sub-nuclear structures called ionizing radiation induced foci (Maser et al., 1997; Pryde et al., 2005; Polo and Jackson, 2011). These foci therefore serve as an excellent physical location in the nucleus to examine roles of different proteins involved in DSB repair. However, this fortuitous natural event of formation of foci that allowed rapid progress in understanding of DSB repair does not occur in UVC-irradiated cells, which prevented progress in study of NER.

The most important breakthrough in understanding mammalian NER came with the simultaneous development of local irradiation technique by two independent groups (Katsumi et al., 2001; Moné et al., 2001). In this method, the cell monolayer is covered with a UVC opaque polycarbonate filter with 3-8 μm pores, permitting irradiation of a small defined area within the nucleus, which can be readily detected by signal for DNA damage in the form of CPD. This technique revealed the order of assembly of NER proteins, ending the long-standing debate that recruitment of all NER proteins to the lesion site depends on the presence of XPC and not XPA (Volker et al., 2001). This technique also laid to rest the hypothesis that mammalian NER is carried out by a repairosome complex since the arrival and departure of GFP-tagged NER proteins, ERCC1-GFP and TFIIH-GFP at the lesion site, occurred as and when they were required in the sequential process of repair (Moné et al., 2004).

Despite its numerous advantages, including the low cost and use of basic equipment, the local irradiation technique needed optimization for the visualization of different repair proteins at damage site in different cell lines (Ticli and Prosperi, 2019). For instance, we observed that the uniform distribution of signal for PARP1 throughout the nucleus before irradiation did not change after local UVC irradiation. This was not due to lack of accumulation of PARP1 at the local site but due to the noise from the strong signal of PARP1 in rest of the nucleus drowning out the minor change in the signal intensity of PARP1 at the lesion site. To circumvent this problem, our team used two independent approaches. First, we used PAR formation as a proxy for recruitment of PARP1 to the lesion site, because binding of PARP1 to any type of DNA lesion results in its catalytic activation and formation of PAR (Pascal and Ellenberger, 2015). Using local UVC irradiation, we showed concurrent signals for CPD and PAR at the site of local irradiation (Vodenicharov et al., 2005). Thus, monitoring the outcome of recruitment of a protein at the local DNA lesion site offered us a good alternative to identify PARP1 as a new player in NER. The second approach was to deplete free PARP1 from nuclei using in situ high salt extraction as an additional step after fixation, to significantly reduce the noise from rest of the PARP1 while retaining the DNA damage-bound PARP1 (Purohit et al., 2016). Similar protocols for selective depletion of unrelated protein molecules using a mild treatment with DNase or RNase have been used to improve the detection of the XPG, DDB2, XPC (Dutto et al., 2017), and Ku80 (Britton et al., 2013) at the lesion sites. Along with this, the live-cell imaging revealed that NER is highly dynamic process with a continuous exchange of the repair factors during the repair reaction (Vermeulen, 2011; Ticli and Prosperi, 2019).

Thus, the local irradiation techniques and its improvements have revealed roles of many proteins in mammalian NER, including the unsuspected implication of the abundant protein PARP1 (Luijsterburg et al., 2012; Pines et al., 2012; Robu et al., 2013). However, local irradiation technique does not work for studying some proteins. For example, it is difficult to visualize accumulation of the GFP-tagged TCR protein CSB at locally induced UVC spots since only a small fraction (15%) of CSB is recruited to the damage site (Van Den Boom et al., 2004; Aydin et al., 2014; Wienholz et al., 2019). While this situation is not different from the abundant protein PARP1, binding of CSB to DNA damage does not result in distinct functional product which could serve as a proxy for CSB recruitment. The second limitation of local irradiation technique with UVC opaque filter is that it allows study of NER only after UVC and not after treatment with other agents, such as cisplatin or Illudin S (Marteijn et al., 2014). Lastly, local UVC irradiation mediated visualization of a given NER protein is not amenable to study how chromatin marks and various PTMs (e.g., ubiquitination, phosphorylation, sumoylation, acetylation, and PARylation) occurring in the vicinity of the damage, affect the speed and accuracy of recruitment of core NER factors (Dantuma and Van Attikum, 2016). These modifications regulate the higher-order structure of chromatin to facilitate the sequential traffic of NER proteins at the lesion site through control over their recruitment and departure as well as their degradation. However, PTMs occur not only during DNA damage response but also for housekeeping functions, and identical PTM occurs on multiple proteins at the same site; hence immunodetection of PTM at local irradiation fails to identify uniquely repair related PTM of a single protein. The immunodetection of proteins after local irradiation would also not discriminate between unmodified and PTM-altered proteins. In this context, the use of sub-cellular and sub-nuclear fractionation linked with immunoprecipitation and immunoblotting approaches modified for a specific protein or its PTM can permit study of the dynamic response of the protein in NER of the DNA damage. The isolation of subcellular fractions (cytoplasmic, nucleoplasmic, and chromatin bound proteins) from mammalian cells has the advantage of revealing the changes in the intracellular redistribution of the proteins of interest after DNA damage. For instance, if PTM of a protein occurs only when it is bound to DNA lesion, then cell fractionation can reveal the enrichment of PTM-modified protein in chromatin-bound fraction. In addition, the co-immunoprecipitation studies of a protein in each fraction can reveal DNA-lesion specific interacting partners of that protein, which could be different from those in other sub-cellular fractions.

Despite the tremendous progress made in the study of mammalian NER, there is need for more studies on multiple fronts in NER. The studies on NER of chemotherapeutic drug-induced DNA damage could reveal clinically exploitable knowledge to improve therapeutic efficacy of these drugs. There could be many other unanticipated proteins, like PARP1, playing different auxiliary roles in controlling the functions of core NER proteins. There are significant gaps in our knowledge of early steps of mammalian TCR sub-pathway, such as roles of other transcription initiation and elongation factors who happen to be present in the vicinity of stalled RNA polymerase II. Lastly, we have just begun to understand TCR of nucleolar DNA at stalled RNA polymerase I, but more studies could reveal an attractive target to control cancer cells which are highly dependent on rDNA transcription to meet demands of fast-growing cells. To stimulate more studies in NER, there is a need for widespread accessibility to techniques that will allow many more laboratories to explore this repair pathway in different models. In this context, some of the leading techniques in the field, such as live cell microscopy imaging using fluorescent tagged proteins (Vermeulen, 2011; Ticli and Prosperi, 2019) or UVC laser with quartz optics to cause damage in defined sub-nuclear zones (Dinant et al., 2007) have produced excellent data and will continue to be useful in future. However, these techniques need specialized reagents, engineered cell lines and expensive instruments which may not be available in most laboratories.

Therefore, here we describe detailed protocols for three relatively simple yet powerful techniques: local irradiation, in situ extraction and subcellular fractionation. These techniques use reagents and tools that are readily available in most laboratories and will allow identification of potential role of both endogenous or exogenous tagged proteins in mammalian NER in most cellular models. These techniques could be readily modified to study NER after treatment with other DNA damaging agents beyond UVC or to specifically study TCR. In addition, the cellular fractionation protocol provides an enriched nucleoplasmic or chromatin-bound protein fraction that can be useful for many downstream applications, such as immunoprecipitation and proteomics to identify the components of repair complexes or partners of the target proteins. Collectively these three simple techniques can provide an initial assessment of the functions of known and unknown proteins in the core or auxiliary events associated with the efficiency of mammalian NER. The results from these techniques could serve as a solid foundation and a justification for more detailed studies in NER using specialized reagents and more sophisticated tools, as required.

## Materials and Equipment

### Reagents for Cell Culture

• Human skin fibroblasts (GM00637-Coriell Institute) or any other cell line of interest.
• Appropriate medium for the cell culture, e.g., Minimum essential medium (αMEM) (Gibco, cat. no. 12561056, store at 4°C).
• Penicillin-streptomycin solution (10,000 U/mL penicillin and 10,000 μg/mL streptomycin, Hyclone, cat no. SV30010, store at −20°C).
• Bovine growth serum (replacement for fetal bovine serum, consists of bovine calf serum supplemented with chemically defined components, Hyclone, cat. no. SH30541.03, store at −20°C).
• Solution of 0.25% Trypsin and 2.12 mM EDTA (Wisent Inc., cat. no. 325-043-EL, store at 4°C).
• 1X Phosphate-buffer saline (PBS), pH 7.4. Prepare 10X buffer containing 1.37 M sodium chloride (NaCl) (Sigma-Aldrich, cat. no. S7653), 27 mM potassium chloride (KCl) (Sigma-Aldrich, cat. no. P9541), 100 mM disodium hydrogen phosphate (Na_2_HPO_4_) (Sigma-Aldrich, cat. no. S0876), and 20 mM potassium dihydrogen phosphate (KH_2_PO_4_) (Sigma-Aldrich, cat. no. P5379) in distillated water. Do not adjust the pH. Store at RT.
• Trypan Blue Stain (0.4%) (Gibco, cat. no. 15250-061).
• PJ34 hydrochloride, PARP1 inhibitor (Abcam, cat.no. ab120981). Prepare a 30 mM stock solution (10 mg/mL) in distillated water. Store the solution as aliquots at −20°C.

### Reagents for Local Irradiation Protocol

• 3% (w/v) Paraformaldehyde solution (PFA) (Sigma-Aldrich, cat. no. P6148; store at 4°C).

Note 1. The PAF solution is prepared fresh prior to use. To prepare 100 mL solution, add 3 g of PFA powder in 80 mL water. To dissolve the PFA, add 100 μl of 1N NaOH and heat the solution in a 60°C water bath. Vortex to mix. Cool the PAF solution, add 10 mL of 10X PBS and 100 μl of 1N HCl solution to adjust the pH at 7.4. Add water to make the final volume to 100 mL and filter it to avoid particles. Keep the solution at RT until use.

*Caution:* PFA is toxic and corrosive. Avoid any direct contact and wear appropriate personal protective equipment.

• C (CSK) buffer. It contains 100 mM NaCl (Sigma-Aldrich, cat. no. S7653), 300 mM sucrose (Sigma-Aldrich, cat. no. 84097), 10 mM PIPES pH 6.8 (Sigma-Aldrich, cat. no. P1851), 3 mM MgCl_2_ (Sigma-Aldrich, cat. no. M2670), and 1 mM EGTA [ethylene glycol-bis (β-aminoethyl ether)-N,N,N’,N’-tetraacetic acid] (Sigma-Aldrich, cat. no. E8145). Prepare the buffer in distilled water. The C buffer can be stored at 4°C for several months.
• 100% Methanol (Fisher Chemical, cat.no. A452-44). *Caution:* Methanol is toxic if inhaled and in contact with skin. Avoid any direct contact and wear appropriate personal protective equipment.
• 100% Trichloroacetic Acid (TCA) (Sigma-Aldrich, cat. no. 490-10). Store at 4°C. *Caution:* TCA is corrosive. Avoid any direct contact and wear appropriate personal protective equipment.
• 100% Ethanol.
• Blocking buffer: PBS containing 5% (w/v) albumin from bovine serum (Sigma-Aldrich, cat. no. A9647) and 0.1% Triton X-100.
• Blocking buffer for PAR detection: PBS containing 5% (w/v) milk powder and 0.1% Tween 20 (Sigma-Aldrich, cat. no. P2287-500 mL).
• 12 N HCl (Sigma-Aldrich, cat. no. 320331-500 mL).
• Wash buffer: 0.1% Tween 20 in PBS.
• DAPI (Sigma-Aldrich, cat. no. D9542) stock solution (5 mg/mL) in distillated water. For long-term storage, the solution can be aliquoted and stored at −20°C. The solution can be keep at 4°C, protected from light, for short term storage. *Caution:* DAPI is mutagen. Avoid any direct contact and wear appropriate personal protective equipment.
• 1X Phosphate-buffer saline (PBS), pH 7.4.
• Mounting solution (Prolong Gold antifade reagent, Invitrogen, cat. no. P36934).
• Primary antibodies: mouse anti-CPD (clone TDM-2, Cosmo Bio, cat. no. NMDND001 used at 1/1000 dilution), mouse anti thymine dimers (T-T) (clone KTM53, Kamiya Biomedical Company, cat.no. MC-062, 1/2000), goat anti-DDB2 (R&D, cat. no. AF3297, 1/500), and rabbit anti-PAR (LP-96-10, Aparptosis, 1/250).
• Secondary antibody conjugated to fluorescent dye: Alexa Fluor 488 and 594 goat anti-rabbit and anti-mouse IgG (Molecular Probe, Invitrogen, cat no. A11029, A11034, A11012, and A11005) and Alexa Fluor 488 donkey anti-goat IgG (Molecular Probe, Invitrogen, cat no. A11055) are used at 1/500 dilution.

### Reagents for *in situ* Extraction Protocol

• 3% (w/v) Paraformaldehyde solution (PFA).
• C (CSK) buffer.
• C+T buffer (CSK buffer + 0.5% Triton): 100 mM NaCl, 300 mM sucrose, 10 mM PIPES pH6.8, 3 mM MgCl_2_, 1 mM EGTA, 0.5% Triton X-100 (Sigma-Aldrich, cat. no. T8787). C+T buffer can be stored at 4°C for several months.
• C+T+S buffer (High salt CSK buffer): 420 mM NaCl, 300 mM sucrose, 10 mM PIPES pH 6.8, 3 mM MgCl2, 1 mM EGTA, 0.5% Triton X-100. C+T+S buffer can be stored at 4°C for several months.
• 100% Ethanol.
• Blocking buffer: PBS containing 5% (w/v) BSA and 0.1% Triton X-100.
• 12 N HCl (Sigma-Aldrich, cat. no. 320331-500 mL).
• Wash buffer: PBS-0.1% Tween 20.
• 5 mg/mL DAPI stock solution.
• 1X PBS, pH 7.4.
• Mounting solution.
• Primary antibodies: mouse anti-CPD (1/1000), mouse anti thymine dimers (T-T) (1/2000), goat anti-DDB2 (1/500), rabbit anti-XPC (Gene Tex, cat. no. GTX70309, 1/1000 dilution), mouse anti-PARP1 (clone F123, Alexis, cat. no. ALX804211, 1/500 dilution), rabbit anti-XPA (Santa Cruz, cat. no. sc-853, used at 1/500 dilution).
• Secondary antibody conjugated to fluorescent dye: Alexa Fluor 488 and 594 goat anti-rabbit and anti-mouse IgG and Alexa Fluor 488 donkey anti-goat IgG (1/500).

### Cell Fractionation Reagents

Note 2. Prepare the buffers in advance without protease or phosphatase inhibitors and PMSF and store it at 4°C and add the inhibitors on the day of the experiment. Alternatively, prepare the complete buffer and freeze it at −20°C.

Note 3. Prepare in advance the protease and phosphatase inhibitors and store in aliquots at −20°C. Thaw them on the day of experiment.

• 1X PBS, pH 7.4. Store at 4°C.
• 100 mM phenylmethylsulfonyl fluoride (PMSF) (Sigma-Aldrich, cat. no. P7626): 17.42 mg/mL in isopropanol.
• 100 mM β-glycerophosphate disodium (Sigma-Aldrich, cat. no. G9422), Ser/Thr phosphatases inhibitor: 21.604 mg/mL (solubility limit 50 mg/mL) in distillated water.
• 1 M sodium fluoride (Sigma-Aldrich, cat. no. S6521), Ser/Thr, and acidic phosphatases inhibitor: 41.99 mg/mL in distillated water.
• 100 mM sodium orthovanadate (Sigma-Aldrich, cat. no. S6508), Tyr and alkaline phosphatases inhibitor: 18.391 mg/mL in distillated water. Set pH to 9.0 with 1N HCl and boil until colorless. Cool to room temperature. Repeat this cycle until the solution remains at pH 9.0 after boiling and cooling. Bring up to the initial volume with distillated water.
• 10X protease inhibitors (Roche, cat. No. 1836170): dissolve one tablet in 1 mL distillated water.
• Buffer A: 10 mM Hepes, pH 7.8 (Sigma-Aldrich, cat. no. H4034), 10 mM KCl, 1.5 mM MgCl_2_, 0.34 M sucrose, 10% glycerol (Sigma-Aldrich, cat. no. G5516), 0.1 % Triton X-100, 1 mM PMSF, 1X protease inhibitor, 10 mM β-glycerophosphate, 10 mM sodium fluoride, 1 mM sodium orthovanadate in distillated water.
• Buffer B: 50 mM Tris pH 7.8 (Sigma-Aldrich, cat. no. T6066), 420 mM NaCl, 1 mM EDTA (Ethylenediaminetetraacetic acid, disodium salt) (Thermo Fisher Scientific, cat. no. BP120-500), 0.5 % IGEPAL (Sigma-Aldrich, cat. no. I3021), 0.34 M sucrose, 10 % glycerol, 1 mM PMSF, 1X protease inhibitor, 10 mM β-glycerophosphate, 10 mM sodium fluoride, 1 mM sodium orthovanadate in distilled water.
• Chromatin extraction buffer: 20 mM Tris-HCl pH 7.5, 100 mM KCl, 2 mM MgCl_2_, 1 mM CaCl_2_ (Sigma-Aldrich, cat. no. C3306), 0.3 M sucrose, 0.1% Triton X-100, 1X protease inhibitor, 1 mM PMSF, 10 mM β-glycerophosphate, 10 mM sodium fluoride, 1 mM sodium orthovanadate in distilled water.
• Micrococcal nuclease solution > 100U/μl (Thermo Fisher Scientific, cat. no. 88216).
• Benzonase solution 379U/μl (Sigma-Aldrich, cat. no. E8263-5Ku).
• 10 mM MG132 (Abcam, cat. no. ab141003) stock solution. Dissolve at 4.76 mg/mL in DMSO. Solution can be aliquoted and store at −20°C.
• 10% TCA solution.
• Solubilisation solution: 0.25N NaOH (Sigma-Aldrich, cat. no. 415413-1L), 0.025% Triton in water.
• 22% Bradford assay dye solution (Biorad, cat. no. 5000006) in distilled water. Store at 4°C.
• Protein (BSA) standard solution (2 mg/mL) (Thermo Fisher Scientific, cat. no. PI23209).
• Antibodies: goat anti-DDB2 (1/1000), rabbit anti-XPC (1/1000), rabbit anti-XPA (used for IP at 1/100 dilution), mouse anti-PARP1 (clone F123, 1/5000), rabbit anti-PARP1 (1/5000), rabbit anti-beclin (Cell Signaling, cat. no. 3495, used at 1/1000), and rabbit anti-histone H3 (Abcam, cat. no. ab1791, used at 1/2000).

### Equipment

• Sterile 35- and 100-mm culture dishes (or other size) (Corning, cat. no. 353001).
• Cell culture incubator (set at 5% CO_2_ and 37°C).
• Hemocytometer (Thermo Fisher Scientific, cat. no. 0267110).
• Microscope cover glass (Thermo Fisher Scientific, cat. no. 12545102).
• Isopore membrane filters (3-8 μm pores, Millipore, cat.no. TMTP02500).
• Forceps.
• Ultraviolet hand lamp EF-140 with UVC lamp (BLE-2537S) (Thermo Fisher Scientific, cat. no. 119921200).
• UVX digital radiometer (UVP, cat. no. 534-243534-89).
• Parafilm (Sigma-Aldrich, cat. no. P76680).
• Inverted microscope (Axiovert 200, Zeiss) with Axiocam MRm camera.
• Microscope slides (Thermo Fisher Scientific, cat. no. 12-550-123).
• 1.5- and 15-mL tubes.
• Pipettes, tips, and scrapers.
• Refrigerated centrifuges.
• Sonic Dismembrator Model 500 (Thermo Fisher Scientific).

## Stepwise Procedures

### Overview

**FIGURE 1:**
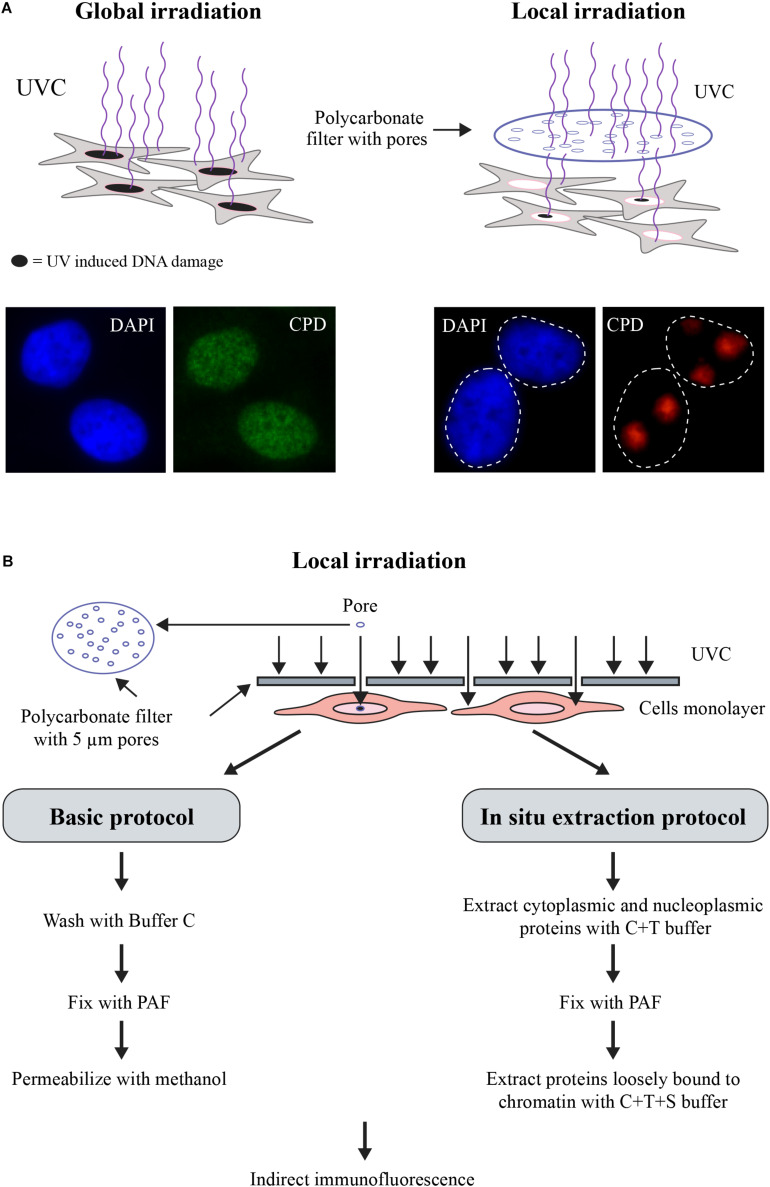
**(A)** Scheme for Global or Local UVC-irradiation. The cells are exposed to UVC either unfiltered for global irradiation (left panel) or through an isopore polycarbonate filter with 3, 5, or 8 μm holes (right panel). DNA damage induced by either protocol of irradiation was detected by indirect immunofluorescence using an antibody specific for UV-induced photolesions. DAPI staining is carried out to define the nuclei. **(B)** Flow chart for the two versions of the local irradiation protocol. In the basic version (left), locally irradiated samples are fixed and processed for immunofluorescent detection, whereas in the *in situ* extraction protocol (right), the locally irradiated samples are processed for removal of non-DNA bound proteins prior to and after fixation step followed by immunofluorescent detection DNA-bound proteins. The steps and the buffer compositions are described in the main text.

**FIGURE 2:**
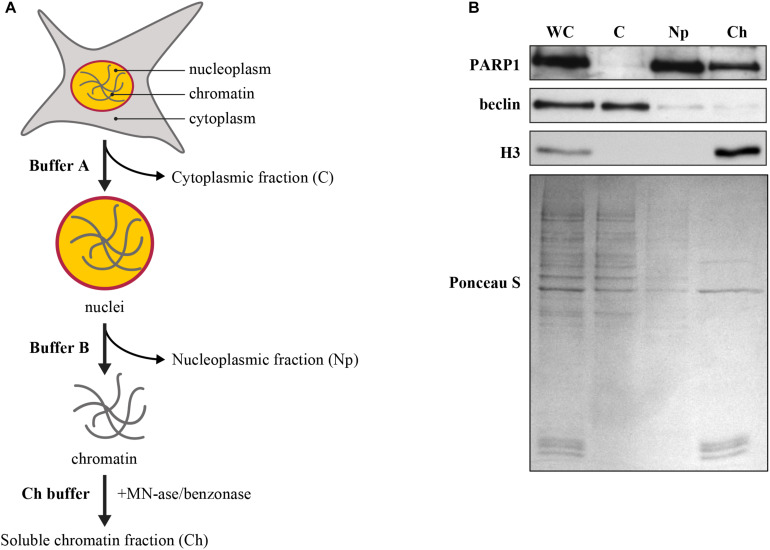
**(A)** Flow-chart for subcellular fractionation. The harvested cells were suspended in appropriate buffer to prepare the whole cell or WC-extract, which was sequentially processed to isolate cytoplasmic or C-fraction (C) and nuclear fraction. The nuclear fraction was further processed to separate nucleoplasm or Np-fraction and chromatin-bound protein or Ch-fraction. The protocol and the buffer compositions are detailed in the main text. **(B)** Validation of subcellular fractionation. The proteins in whole cell (WC), cytoplasm (C), nucleoplasm (Np), and chromatin bound (Ch) protein fractions were resolved by SDS-PAGE, transferred to nitrocellulose membrane and probed for specific markers for each of the cellular compartments, such as PARP1 for entire nucleus (nucleoplasm and chromatin), histone H3 for chromatin alone, and beclin for cytoplasm. Ponceau S staining was used as loading control.

Protocols described here have been widely used in NER field for the last 20 years (Ticli and Prosperi, 2019). They have been adapted in our laboratory for analyses of PARP1 accumulation and its activation to form PAR at the DNA lesion site and its influence on the movement of NER proteins after induction of DNA damage. A scheme for each of these protocols is shown in Figures 1, 2. Cells are processed in monolayer after local irradiation with UVC (Figure 1) to visualize the movement of target proteins using immunofluorescent labeling. To improve the detection signal of the repair proteins at the lesion site, the unbound or “free” cytoplasmic and nucleoplasmic proteins are extracted by submerging the coverslips in buffers with increasing detergent and salt concentrations. Globally irradiated cells are either scrapped off the dishes or trypsinized to form single cell suspension (Figure 2A) and centrifuged followed by sequential extraction of the cell pellet in different buffers. The liberated cellular fractions are collected by centrifugation at each of the steps (Figure 2A). After removal of the nucleoplasm, the chromatin pellet is digested with high concentration of nucleases (MNase or benzonase) to liberate chromatin-bound protein fraction. The movement of repair proteins is analyzed by migrating these fractions on SDS-PAGE gel followed by immunoblotting of various proteins (Figure 2B).

#### A. Stepwise Procedure for Basic Local Irradiation Protocol

##### A.1 Cell culture [approximately ∼ 24 h before irradiation]

A.1.1 Sterilize the glass coverslips. With sterile forceps, dip the glass coverslips in 100% ethanol. Air dry by place them in an angle into a 15 cm plate. Once they are dry, they are placed in 35 mm sterile dishes.

Note 4. Prepare one coverslip for each experimental time point and controls. To determine the number of samples, one needs to understand that the normal NER kinetics at damage site (15 s to 3 h) is affected by gene manipulation. For example, the XPC half-life at damage site is 1 h, however, in XPA deficient cells, the level of XPC was stable from 30 min to the end of protocol (3.3 h) (Luijsterburg et al., 2010). Hence, it is very important to run a pilot experiment in which early and late time points are included.

Note 5. The buffers volumes are for one 35 mm dish.

Note 6. Certain controls need to be run with each experiment, so plan to include these conditions when planning to seed the cells: IgG in place of primary antibody to check the specificity of the primary antibody, secondary antibody alone without primary antibody to verify lack of non-specific binding and no antibodies to verify autofluorescence.

A.1.2 In order to prepare cells on coverslips, calculate the number of coverslips needed for the experiment and the total number of cells required. Scale up the cells to have 30-40% more cells than that required on the day of seeding the coverslips. Trypsinize the cells and take a cell count to ensure that there are enough cells to carry out the experiment and that the number of cells seeded are same in each experiment. In our experiments, we add 300,000 cells per dish in 1.5 ml medium. If we need to seed 10 dishes, we make a master mix for about 13 dishes, by suspending 300,000 × 13 (3.9 × 10^6^) cells in total of 1.5 × 13 ml (19.5 ml) medium and we add 1.5 ml of this slowly on the glass coverslips. Before each pipetting, mix the medium well. Cells should be at 70-80% confluency at the time of treatment, however this can vary depending on the experimental setting.

A.1.3 Grow the cells on the coverslip for 24 h in appropriate condition (5% CO_2_, 37°C).

##### A.2 UVC irradiation [timing ∼ 2 min per plate]

A.2.1 Pre-warm the UVC lamp for at least 10 min to avoid dose variability between the samples.

A.2.2 Measure the lamp flux using the UV meter across the surface of the irradiation. Determine the surface area required to irradiate based on the size of the dish used for the experiment. Measure the doses in five spatial points (the four corners and the middle of the irradiation surface) of this region and calculate the average to determinate the time needed to obtain certain dose of UVC, knowing that Dose = Intensity of the lamp (mw/cm^2^) × Time of exposure (sec). Place the dish within this region to accurately irradiate the cells.

A.2.3 Aspirate the media or remove it and store it for reuse. Wash the cells one time with 2 mL of 1X PBS. Aspirate the PBS and add 500 μl 1X PBS. For global irradiation, go to step A.2.4. For local irradiation, gently place the polycarbonate filter having 5 μm pores over the cell monolayer. Slowly aspirate the PBS from the dish. This will leave just enough PBS to form a thin layer between the cells and the filter.

Note 7. Do not move the filter once placed because of the risk to detach the cell-layer.

Note 8. The UVC rays do not pass through plastic, hence the lid of the dish is removed, but only once the dish is placed inside the UV chamber, to avoid cell contamination. To irradiate the cells, remove the lid of the dish and start the timer simultaneously. After the calculated time, place the lid back to stop irradiation.

A.2.4 Irradiate the cells with a dose of 100 J/m^2^ UVC for local irradiation or 10 J/m^2^ (or specified dose) for global irradiation. Doses are applied by increasing the time of exposure.

A.2.5 Add media back to the dish and incubate for an appropriate time depending on the experiment setup.

##### A.3 Cell fixation and permeabilization [timing ∼ 1 h]

Note 9. Do not allow the cells to dry out during any steps, since drying will increase the background fluorescence. When a buffer is aspirated, the new buffer should be added quickly.

Note 10. To stain the polymers of ADP-ribose, follow the steps A.3.6 to A.3.9.

A.3.1 Aspirate the media and wash 2 times with 1.5 mL of buffer C.

A.3.2 Fix the cells in 1.5 mL of 3% paraformaldehyde for 10 min at RT.

A.3.3 Wash 3 times with 2 mL 1X PBS.

A.3.4 Permeabilize in 1.5 mL of cold 100% methanol for 30 min at −20°C.

A.3.5 Wash 2 times with 2 mL 1X PBS.

A.3.6 Aspirate the media and wash with 1.5 mL of cold PBS.

A.3.7 Fix the cells in 1.5 mL of 10% TCA in PBS for 15 min on ice.

A.3.8 Aspirate the TCA and add 1.5 mL of cold 70% ethanol; incubate for 3 min at RT.

A.3.9 Repeat the step A.3.8 using sequentially 90 and 100% cold ethanol.

##### A.4 Indirect immunofluorescence [timing ∼ 4 h]

A.4.1 Incubate the coverslips in 1.5 mL blocking buffer for 30 min at RT or overnight at 4°C. These steps will prevent non-specific antibody binding. *Safe stop!* The coverslips can be stored at 4°C for 3-4 days in the blocking solution. In this case, seal the plate with parafilm strip around the lids to avoid evaporation and sample drying.

A.4.2 *Optional!* Immunofluorescent labeling of CPD or 6-4PP in UV-damaged DNA requires denaturing of cellular DNA. To do this, after blocking, wash the coverslips 5 times with PBS and incubate them for 5 min at RT in 1.5 mL of 2 N HCl.

Note 11. During this incubation time, prepare a humidified chamber by placing a moist paper towels at the bottom of a box or dish. Cover the paper with parafilm, so that the coverslips are not in direct contact with the paper towels. Since the coverslips will be placed on it, information necessary to identify the coverslip can be written on the parafilm using a fine permanent marker (do this at regular spacing, taking into account the size of the coverslip, such that the coverslip can be placed without touching each other).

A.4.3 Wash 5 times with 2 mL 1X PBS.

Note 12. Do not aspirate the last PBS wash. Removing coverslip from a bufferless dish results in their breaking due to surface tension. After removing the coverslips, do not discard the dishes. The coverslips are placed in their respective dishes for washings between the antibody incubations.

A.4.4 Dilute the first antibodies in blocking buffer (40 μl of diluted antibody per 25 mm coverslip). Place 40 μl drops of the diluted antibody near each identified spot on the prepared parafilm. Remove the coverslip from the dish using pointed forceps and blot the excess buffer by touching the edges of the coverslips on a paper towel. Gently invert the coverslip over the antibody, placing it “cell side facing down” over the drop. Cover the box with a lid or an aluminum foil to keep the moisture in. Incubate for 1 h at RT or overnight at 4°C.

A.4.5 Using the same forceps, gently remove the coverslip from the humid chamber and place it “cell side facing up” in its original dish containing 2 mL of wash buffer.

Note 13. Be very careful to not mix up the side on which the cells are grown.

A.4.6 Wash with 2 mL of wash buffer 3 × 5 min each.

Note 14. Subsequent steps must be done in the dark to minimize the photobleaching of the fluorophore. Cover the dishes containing the coverslips and the humidified chamber with aluminum foil.

A.4.7 Dilute the secondary antibody at 1/500 dilution in blocking buffer. Proceed as describe in Note 11, 12 and step A.4.4. Incubate the coverslips in a humidified chamber for 30 min at RT.

A.4.8 Transfer the coverslips back to the dishes and wash with 2 mL of wash buffer 3 × 5 min each.

A.4.9 Prepare 1.5 mL of DAPI solution (0.25 μg/mL) in 1X PBS per 35 mm dish. Add over the coverslip in the plates and incubate for 5-10 min at RT. DAPI helps in microscopic identification of the nuclei by staining the DNA.

A.4.10 Wash 2 times with 2 mL 1X PBS to remove excess DAPI.

A.4.11 Wash 2 times with 2 mL distillated water to remove salts.

A.4.12 Add 2 mL of distillated water in the dish and remove the coverslips with forceps. Remove the excess water by dabbing the edges of the coverslips over a paper towel. Let the coverslips air dry into a box on a paper towel.

A.4.13 Mount the coverslips on a microscopic slide by inverting them on a drop of mounting media. The 20 μl drop of mounting media is placed on a slide using a 200 μl tip with a cut end. Take care to not introduce air bubbles while placing the drop and while placing the coverslip over it. Allow the antifade to slowly spread and cover the entire coverslip with minimal leakage outside the border of the coverslip. Seal the coverslips edges with clear nail polish to prevent drying and movement of the coverslips during image acquisition.

*Safe stop!* The slides can be viewed immediately or stored at −20°C, in dark, for months.

##### A.5 Image acquisition and analysis [timing ∼ 2 days]

Note 15. An important issue in interpreting the results of the recruitment of protein in the damaged region of the nucleus is the quantification of the signal. In the local irradiation technique, since most of the NER proteins would be present in the nucleus even before irradiation, the extent of enrichment of the protein at the site of DNA lesion after irradiation is derived as fold increase in signal intensity at the lesion site that is identified by CPD signal over the signal for the same protein in equivalent area of interest in the portion of the nucleus that is not irradiated. To analyze hundreds of images of this type and obtain a very robust statistical data in shorter time, it is advisable to develop macros either for the microscopy software or freely available Image J program.

A.5.1 Take the images of a least 100 nuclei at 40x or 63x magnification. We use an inverted fluorescence microscope Axiovert 200 (Carl Zeiss) equipped with AxioCam MRm.

*Safe stop!* Data processing can be conducted as per convenience of the user.

A.5.2 Delineate manually all the CPD positive spots using AxioVision 4.7 or others type of image analysis software.

A.5.3 To quantify the level of protein at lesion site, measure its fluorescence at the CPD spots.

A.5.4 Delineate and measure the intensity of a similar area in the unirradiated zone of nucleus to obtain a background corrected signal for the protein of interest.

A.5.5 Subject the data for the intensity of at least 100 spots from three independent experiments to statistical analysis to determinate the significance of difference.

#### B. Stepwise Procedure for *in situ* Extraction Protocol

Note 16. Cells are seeded and irradiated as described in local irradiation protocol; follow the steps A.1 and A.2.

##### B.1 Cell fixation and permeabilization [timing ∼ 1 h]

Note 17. Do not allow the cells to dry out during any steps, since drying will increase the background fluorescence. When a buffer is aspirated, the new buffer should be added quickly.

B.1.1 Aspirate the media and wash 2 times with 1.5 mL of buffer C.

B.1.2 Permeabilize in 1.5 mL of buffer C+T for 8 min at RT. This step will remove soluble cytoplasmic and nucleoplasmic proteins.

B.1.3 Wash 2 times with 2 mL 1X PBS.

B.1.4 Fix the cells in 1.5 mL of 3% paraformaldehyde for 10 min at RT.

B.1.5 Wash 3 times with 2 mL 1X PBS.

B.1.6 Extract with C+T+S buffer for 20 min on ice to remove the protein bound to chromatin under unchallenged condition. This step is *optional* and recommended to extract the abundant proteins bound to undamaged chromatin.

B.1.7 Wash 3 times with 2 mL 1X PBS.

##### B.2 Indirect immunofluorescence [timing ∼ 4 h]

Note 18. Follow the step A.4 described in local irradiation protocol.

##### B.3 Image acquisition and analysis [timing ∼ 2 days]

Note 19. Follow the step A.5 described in local irradiation protocol.

#### C. Stepwise Procedure for Subcellular Fractionation Protocol

Note 20. Before starting the procedure, prepare all your solutions and keep them refrigerated. Switch on the refrigerated centrifuges. Perform all steps on ice to avoid proteins degradation. When scraping the cells from the plates, place them on ice (make a thin layer of ice in trays and place aluminum foil on it. Place the plates on these ice trays). Identify and prepare all the tubes for the cell collection and place them on ice.

Note 21. Although the volumes are small, use 15 mL tubes and a centrifuge with a swing out rotor to obtain good pellets.

Note 22. Seed at least 10 cm dish per condition. Cell should be at 80% confluency the day of irradiation. The buffer volumes are for one 10 cm petri dish.

Note 23. Irradiate the cells globally with 10 J/m^2^ UVC by following the steps described in A.2, except step A.2.3 (without polycarbonate filter).

Note 24. If using proteasome inhibitors to study protein degradation due to ubiquitination, the proteasome inhibitor MG132 is added at 5-10 μM to the cells, 1 h before treatment.

##### C.1 Extraction of soluble protein fractions [timing ∼ 3 h]

C.1.1 Aspirate media and wash the plate once with 3 mL cold 1X PBS. Scrape the cells in 1 mL cold PBS and transfer them to a 15 mL tube placed on ice. Add another 1 mL cold PBS to the dish, scrape and pool with the previously scraped cells.

C.1.2 Centrifuge at 600*g* for 10 min at 4°C.

C.1.3 Aspirate the PBS and keep the tube on ice.

C.1.4 Add 190 μl of cold buffer A to the cell pellet. Mix with P200 pipette several times and leave the tube on ice for 7 min. Remove an aliquot as whole cell extract (WC) (about 30 μl to which add 30 μl of 2X loading buffer).

C.1.5 Centrifuge the extract at 1000*g* for 5 min at 4°C.

C.1.6 Transfer the supernatant using a P200 to labeled Eppendorf tube. This is the cytoplasmic extract. Take an aliquot for Western (30 μl).

C.1.7 Wash the pellet once with 190 μl of buffer A; mix using P200.

C.1.8 Centrifuge the extract at 1000*g* for 5 min at 4°C.

C.1.9 Discard the washing (remove with P200, taking care not to touch the pellet).

C.1.10 Add 190 μl of buffer B to each tube, mix with P200 pipette and transfer the cells to Eppendorf tubes. The extracts should be viscous due to nuclear breakage and release of DNA.

C.1.11 Incubate the tube on ice for 30 min and centrifuge at maximum speed (16,000*g*) at 4°C for 30 min.

C.1.12 Transfer the supernatant into a new tube; this is the nucleoplasmic extract. Take a fraction for western (30 μl). Keep the pellet to extract chromatin.

*Safe stop!* You can either extract the chromatin on the same day or freeze the pellet and extract it later. Freeze all others fractions at -20°C.

##### C.2 Extraction of chromatin bound protein fraction [timing ∼ 2 h]

Note 25. The chromatin bound proteins are removed by digestion with nucleases in chromatin buffer. It is important to optimize the removal of proteins bound to chromatin in the cell extracts. This can be done by resolving the DNA released after nuclease digestion on agarose gel.

Note 26. To prepare the chromatin-bound protein fraction, we use 100 U/mL MNase or 25 U/mL benzonase. Higher amount of MNase (4000 U/mL) or benzonase (50 U/mL) can be used to digest the DNA down to mononucleosomal level.

C.2.1 Suspend the chromatin pellet in 50 μl of chromatin buffer.

C.2.2 The tight chromatin pellet is opened by mild sonication at lowest setting (setting 11 in Sonic Dismembrator Model 500 from Thermo Fisher Scientific) for 10 s. For higher volumes, time might be more, up to 15-20 s. If the clumps of chromatin persist, put the tube on ice after the first sonication, and then sonicate again, for 10 s. Usually one sonication is enough to take care of the clumps.

C.2.3 Add 100 U/mL MNase to each tube (0.25 μl of 20 U/μl stock). Mix by vortexing. Incubate the tubes at RT for 40 min. Vortex intermittently.

C.2.4 Stop the reaction with 5 mM EDTA (1 μl of 500 mM stock solution) and 5 mM EGTA (5 μl of 100 mM). Spin at maximum speed for 10 min and collect the supernatant into a new tube. This is the chromatin extract.

*Safe stop!* You can either freeze the fraction at −30°C or proceed to the Western blot analysis.

##### C.3 Western blots analysis of cellular fractions [timing ∼ 2 days]

Note 27. Always validate the protocol by verifying the purity of different subcellular compartments before analyzing the movement of repair proteins, since many of them are present in more than one cellular compartment at the time.

Note 28. Cell fractions are run on Western blots either based on their cell numbers or protein content. Protein estimation is done by using Bradford assay in a 96 well plate.

C.3.1 Whole cell extract in C.1.4: Sonicate the whole cell extract fraction at setting 45 for 20 seconds to reduce viscosity. This can be either frozen for later use or used to estimate proteins followed by Western blot analysis right away.

C.3.2 For protein estimation in WC, precipitate the proteins by adding 1-2 μl of the above prepared extract to 100 μl of cold 10% TCA on ice for at least 30 min. Centrifuge at 16000 g for 5 min. Wash the obtained protein pellet with ethanol to remove traces of TCA and dissolve it in 50 μl solution of 0.25N NaOH-0.025% Triton X-100.

C.3.3 To estimate the proteins in nucleoplasmic and chromatin extract obtained in C.1.12 and C.2.4 dilute the fractions 1: 20 and 1:10 in NaOH-Triton X-100 buffer, respectively. This will prevent the interference of salt and detergents on protein estimation and also bring the concentration of the sample in the linear range of standard curve for protein estimation (5-150 μg/mL).

C.3.4 Quantify the protein concentration using a Bradford assay or similar assay.

C.3.5 Separate 5-10 μg proteins of each cellular compartment on 10 and 12% SDS-PAGE, transfer it in wet condition (100V for 90 min or 35V for 16 h) in Tris-glycine transfer buffer without SDS to a nitrocellulose membrane (GE Healthcare Life Science) and probe with primary antibody against known cytoplasmic (beclin 1/1000), nuclear (polyclonal PARP1 1/1000), and chromatin bound protein (H3 1/2000) markers (Figure 2B). We observe the localisation of beclin in cytoplasm, that of histone H3 on the chromatin, whereas PARP1 is present in both, nucleoplasm and chromatin-bound fraction.

## Results and Discussion

The three simple methods described above do not require specialized reagents and equipment and can be readily performed in most laboratories. These methods will permit analyses of the fate and functions of various endogenous or exogenous proteins during NER. These functions range from recruitment and persistence or departure from the lesion, their interactions with other partners and role of PTMs in these processes. If one uses exogenous tagged NER proteins, note that PTM may be affected by the presence of tag, as observed for DDB2 and XPC (Puumalainen et al., 2014; Matsumoto et al., 2015), which will require validation with untagged or endogenous proteins. The fluorescent signal from tags, such as GFP may be quenched by some solvents or chemicals used in these protocols, therefore, an antibody-based detection of the tag or the protein may be required. Here, we provide examples of how these protocols could specifically reveal the NER-related functions of four key proteins DDB2, PARP1, XPC and XPA in GM human skin fibroblasts.

### Local UVC Irradiation With Immunofluorescence Detection as a Primary Screen to Identify Proteins Recruited to DNA Lesion During NER

**FIGURE 3:**
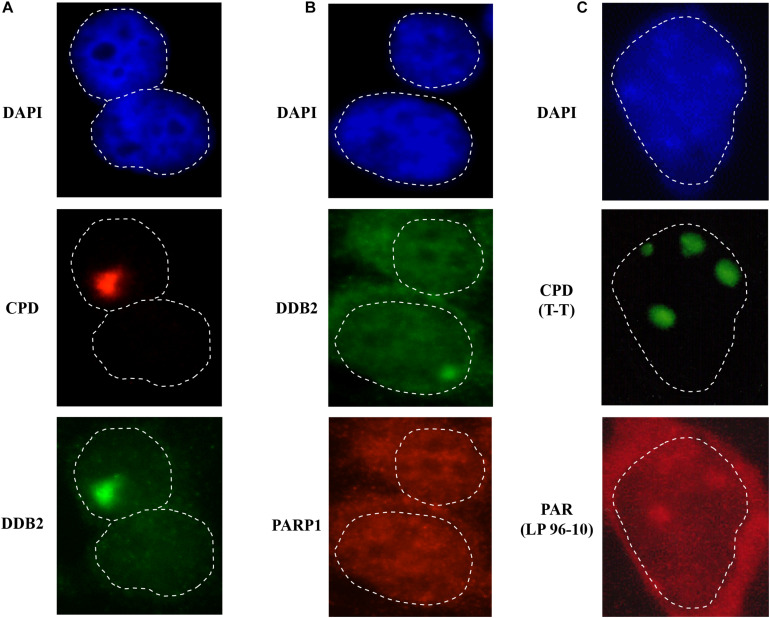
Detection of early NER proteins at the site of damage. GMU6 human skin fibroblasts were locally irradiated with 100 J/m^2^ through 5 μm pores filter, fixed after 10 min and processed for immunofluorescence labeling for CPD and DDB2 (panel **A**) or DDB2 and PARP1 (panel **B**). **(C)** PAR formation at local damage site. GMU6 were fixed with TCA-ethanol at 15 sec after local irradiation and probed for PAR and CPD using the specific antibodies. DAPI staining was carried out to define the nuclei. Note that the cytoplasmic leakage of signal for proteins or PAR (panels **B,C**) is caused by use of the harsh chemicals, such as TCA for PAR-precipitation or methanol for membrane permeabilization. Nonetheless, the signal for these targets immobilized at lesion site remains clearly visible in the nucleus.

The local irradiation of cells at 100 J/m^2^ UVC through an isopore polycarbonate filter with irregularly distributed 5 μm holes (Figure 1) results in DNA damage in defined subnuclear regions (Katsumi et al., 2001; Moné et al., 2001). Note that local irradiation requires higher dose than global irradiation to achieve comparable levels of DNA damage because most of the incident UVC-irradiation reaching the cells from different angles of the source lamp is blocked by UVC-opaque filter except the radiation coming directly from above the pores. The cells can be harvested at various time points after irradiation to follow the time course of recruitment of proteins at damage site. After harvesting, cells are fixed with formaldehyde to preserve cellular morphology, immobilize cellular components in their original locations and prevent their degradation. Since antibody molecules are too large to enter the cells, the fixed cells are permeabilized by treatment with non-ionic detergents such as Triton X-100 (T) followed by direct or indirect immunocytochemistry protocols to detect the proteins of interest in the locally irradiated zone versus their native distribution in the nucleus and/or in the cytoplasm. Using this pioneering technique, Volker et al. (2001) clearly showed the sequential accumulation of key endogenous NER proteins XPC, XPB, and XPA to the local UV-irradiated sub-nuclear zones. Here, as an example, we show the enrichment of the signal for endogenous DDB2 (green signal), a protein arriving rapidly at the subnuclear UV-lesion site containing CPD (red signal), within 10 min after local UVC irradiation (Figure 3A). DDB2 has been shown to facilitate recruitment and stabilization of XPC at the lesions site (Puumalainen et al., 2016). The basic local UVC irradiation readily serves as a low cost and relatively simple technique for primary screening of recruitment of various proteins to the DNA lesion site. Once the recruitment of any protein to lesion site is confirmed, one could perform more detailed studies to understand its significance in NER.

However, one of the limitations of local irradiation technique is that it does not work for proteins that are abundant in the nucleus, such as PARP1. In an unchallenged condition, PARP1 is free in the nucleoplasm and also bound to the chromatin for its housekeeping functions (Kim et al., 2005), hence it exhibits a homogenous distribution throughout the nucleus. Since a very small fraction of PARP1 localizes at DNA damage after local UVC-irradiation, it is challenging to discern this minor enrichment of the signal of PARP1 at the DDB2 spots (which serve as proxy for DNA lesion site) from the overwhelming background signal from rest of the nuclear PARP1 (Figure 3B). To circumvent this problem, we exploited the fact that after binding to any type of DNA damage, PARP1 is strongly catalytically activated to form PAR (Pascal and Ellenberger, 2015). Hence, we used PAR detection as a proxy for recruitment and binding of PARP1 at the site of DNA lesion (Vodenicharov et al., 2005). Using local UVC irradiation, we showed that signal for PAR colocalizes with CPD within seconds at the site of UV-lesion (Figure 3C). It is important to note that strong reagents used for fixation or permeabilization of cells, such as use of methanol with formaldehyde (for DDB2 and PARP1) and TCA (for PAR), can affect the integrity of nuclear structure thereby releasing the nuclear content in the cytoplasm (Hoetelmans et al., 2001), which is visible in local irradiation protocol (Figure 3C). Nonetheless the fractions of DDB2 or PAR linked to DNA remain clearly visible in this protocol. The rapid formation of PAR within seconds after UVC irradiation (Vodenicharov et al., 2005) when CPD are just formed and DNA strand breaks by NER have not yet been created, challenged the prevalent opinion that PARP1 could not play any role in NER, which was based on two assumptions, namely PARP1 activation requires DNA strand breaks and PARP1 is not a member of the repairosome complex. Subsequent studies from many teams, including ours, showed that rapid arrival of PARP1 and PAR formation at the CPD lesion site play key roles with DDB2 to initiate GGR and to recruit XPC to the lesion site (Luijsterburg et al., 2012; Pines et al., 2012; Robu et al., 2013, 2017). The time course of local irradiation also showed that PARP1 activation at the lesion site is a transient process because the signal for PAR disappears within one hour after irradiation (Vodenicharov et al., 2005). Thus, local UVC irradiation is a simple technique that can be used in any cellular model for primary screening of the direct or indirect role of any protein or a process in NER of UVC-induced DNA damage.

### Local Irradiation With *in situ* Cell Fractionation as a Sensitive and Quantitative Method to Assess Enrichment of Proteins to DNA Lesion During NER

**FIGURE 4:**
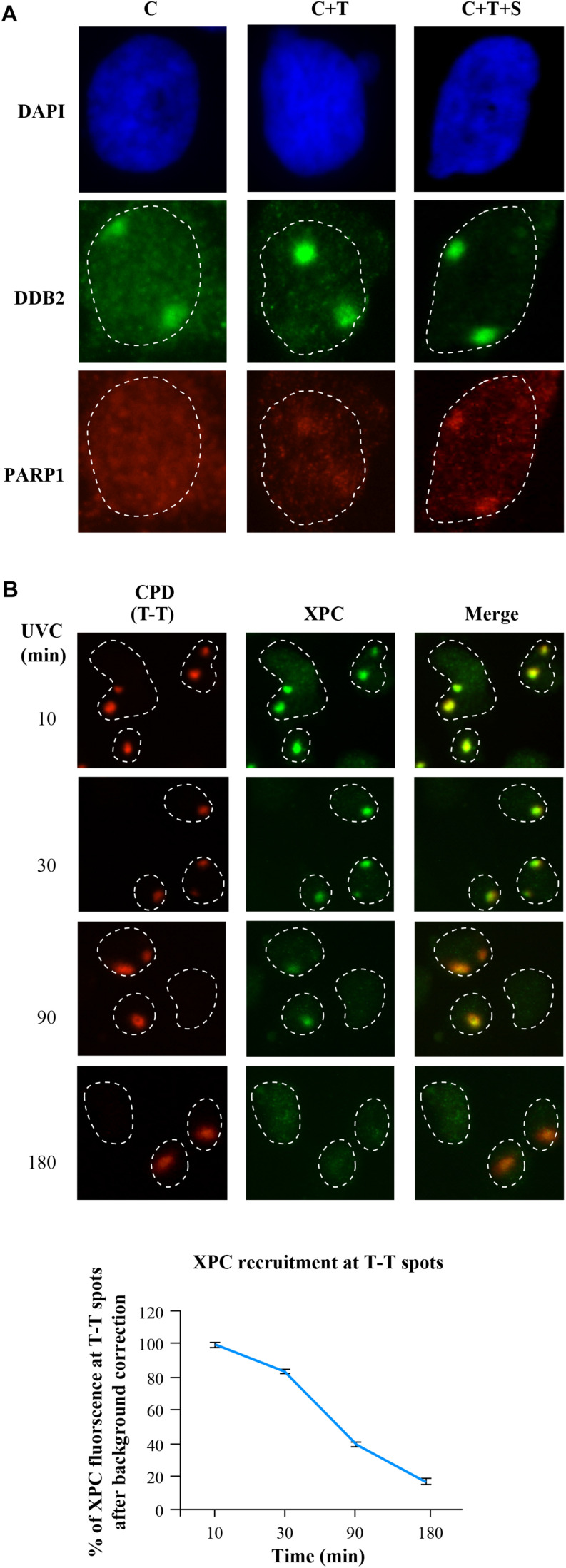
*In situ* extraction for enhancement of signal for NER proteins DDB2, PARP1 and XPC at DNA damage site after local UVC irradiation. **(A)** GMU6 cells were irradiated locally with 100 J/m^2^ UVC and after 10 min subjected to the three protocols (C, C+T, and C+T+S). The immunofluorescent visualization of DDB2 and PARP1 in DAPI stained nuclei improved significantly after each step of in situ extraction protocol. **(B)** The kinetics of XPC accumulation at CPD site after local irradiation was measured at the indicated time after C+T extraction protocol. Data from a least 100 T-T spots from 3 independent experiments were pooled and expressed as percent of the XPC signal at 10 min (100%). DAPI staining was carried out to define the nuclei.

The basic local UVC irradiation technique does not work for direct detection and quantification of the recruitment of the abundant nuclear proteins at the damage site, because the strong signal from rest of the protein would mask the minor extent of protein enrichment at the lesion site. In case of PARP1, we used proxy signal of PAR formation at the lesion site to circumvent this problem, but this proxy option is not feasible for most of the NER proteins. Moreover, PAR can be made by other PARPs too, and therefore, it would be ideal to directly demonstrate the recruitment of PARP1 to the lesion site. Hence, here we describe a method that offers a more general solution by combining local irradiation with the selective depletion of the unbound or “free” proteins from the nuclei (noise) to reveal the signal for lesion-recruited proteins (Figure 4), This method allowed us to detect and quantify the extent of enrichment of PARP1 at the lesion site (Purohit et al., 2016). Since Triton removes free proteins not bound to DNA, the CSK buffer containing 100 mM NaCl (buffer C) with Triton X-100 (C+T) has been used prior to fixation with formaldehyde in the immunofluorescence detection of repair proteins recruited to damaged DNA (Balajee and Geard, 2001; Mirzoeva and Petrini, 2001). Almost all the DDB2 signal outside the local irradiation zone was depleted in C+T sample, demonstrating a clear improvement of signal to noise ratio for DDB2 in C+T buffer over C buffer after local irradiation (Figure 4A, DDB2 panel). However, C+T did not significantly deplete PARP1 protein outside the local irradiation zone. It was earlier shown that 1.6 M NaCl could deplete all the PARP1 from cells (Kaufmann et al., 1991). Therefore, we titrated the salt concentration to determine that 0.42 M salt added to C+T buffer to create (C+T+S (salt)) buffer can extract most of the unbound PARP1 as well as DDB2 from unirradiated cells or from the unirradiated zones of the nuclei. With C+T+S protocol, we could clearly visualize recruitment of PARP1 to the local irradiation zone and also improve the signal to noise ratio for DDB2 (Figure 4A, DDB2 and PARP1 panels).

The improved protocol not only permitted quantification of PARP1 enrichment at the UVC-lesion of irradiated cells but also identified its localization to DNA for housekeeping functions in the unirradiated control cells (Purohit et al., 2016). We also used this improved technique to obtain kinetics of recruitment and departure of XPC protein over 3 h after local UVC irradiation (Robu et al., 2017). This was achieved by quantification of the extent of enrichment of XPC at the lesion site over the background signal for XPC in non-irradiated zone of the nucleus (Figure 4B). Here, we noted a strong accumulation of XPC at CPD lesions at 10 min after irradiation followed by a steady decline by 180 min (Figure 4B and chart), confirming the previously published data (Wang et al., 2003; Puumalainen et al., 2014). Together, the basic local irradiation combined with in situ extraction by C+T or C+T+S buffers can readily permit immunofluorescent visualization and quantification of the signal of a much larger group of NER proteins at the site of local UVC-damaged DNA.

### Sub-cellular Fractionation After Global UV Irradiation to Study Diverse Functional Aspects of NER Proteins

The basic and improved local UVC irradiation techniques allow visualization of recruitment of NER proteins to the lesion site. Here, we describe an alternative approach of the global UVC-irradiation followed by sub-cellular fractionation and immunoblotting to examine biochemical and mechanistic studies related to recruitment, tracking intracellular movement of NER proteins and their interaction with other partners in different cellular compartments. This technique is suitable for study of NER of DNA damage caused by global UVC irradiation, as described here, but also by chemical agents or drugs in cellular models. In this protocol, it is important to incorporate multiple validation steps during the experiments to minimize the variability in data and to generate the quantitative data from Western blot analysis. These validation steps include quantification of protein extracts, validation of antibody and internal controls, detection of the combined linear range for the target protein for the detection methods and internal loading control (Taylor and Posch, 2014). We have already used this technique to identify the role of PARP1 in the lesion recognition step of NER pathways (Vodenicharov et al., 2005; Robu et al., 2013, 2017; Purohit et al., 2016). Here, we describe various uses of sub-cellular fractionation technique to study different functional aspects of NER proteins.

#### Kinetics of Recruitment and Departure of Repair Proteins During NER

**FIGURE 5:**
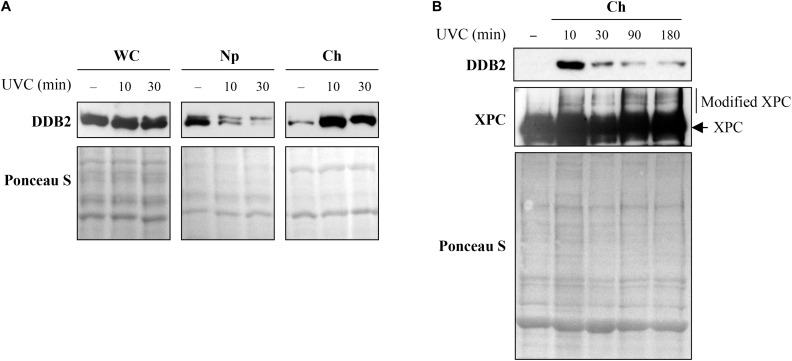
Sub-cellular and sub-nuclear fractionation of globally UVC-irradiated cells to identify movement and localization of NER proteins to UV-damaged chromatin. The GMU6 cells globally irradiated with 10 J/m^2^ were fractionated and subjected to Western blot analysis. Ponceau S staining was used as control for loading. **(A)** The sub-cellular fractions were probed for DDB2 to demonstrate its significant time-dependent depletion in the nucleoplasm and concomitant accumulation in the chromatin-bound protein fraction signifying its movement from inside the nucleus to damaged site on the chromatin in response to global UV-irradiation of the cells. **(B)** Kinetics of arrival and departure of DDB2 and treatment-dependent post-translational modification of XPC in the chromatin-bound protein fraction extracted at different time points after global UVC irradiation. Ponceau S staining was used as loading control.

To assess the timing of initial recruitment and accumulation of early NER proteins at the UV damage, cells were harvested before or 10 and 30 min after global irradiation with 10J/m^2^ UVC to obtain whole cell (WC), nucleoplasm (Np), and chromatin-bound (Ch) protein fractions. Each of these subcellular fractions were separated on SDS-PAGE and immunoblotted for DDB2 (Figure 5A). As shown in earlier studies (Matsumoto et al., 2015), a significant amount of the cellular DDB2 (WC fraction) was present in the nucleoplasm (Np) but not in the chromatin-bound fraction (Ch) prior to irradiation. However, 10 min after irradiation, there was a massive accumulation of DDB2 in the chromatin fraction accompanied by its depletion from the nucleoplasmic fraction, confirming this observation made earlier (Rapic-Otrin et al., 2002). Note that the Western blot of whole cell extract (WC) reveals no change in the signal for DDB2 after irradiation. Thus, the sub-nuclear fractionation of cells at several time points after global UVC irradiation can serve as a relatively simple primary screen to characterize the movement of some NER protein from different cellular compartments (cytoplasm to nucleoplasm) to the UV-induced DNA lesions in the chromatin.

However, this simple sub-nuclear fractionation approach does not work for all NER proteins. For example, when we tracked chromatin-bound DDB2 and XPC for up to 3 h after global UVC irradiation, we observed a strong accumulation of DDB2 at 10 min and a significant decrease from 30 min to 3 h (Figure 5B, DDB2 panel). In contrast, we could not identify any significant change in XPC levels from 10 min to 3 h after irradiation (Figure 5B, XPC panel), which has been reported earlier (Fei et al., 2011). However, it was evident from the local UVC irradiation linked to situ fractionation studies that XPC was indeed recruited to the lesion site 10 min after irradiation and departed by 3 h (Figure 4B). This difference in results between two techniques could be because the lesion recognition proteins, such as XPC, constantly scan the DNA for the presence of damage by association and dissociation from chromatin, which explains large signal for chromatin-bound XPC in control cells. Moreover, upon UV irradiation, while most of the cellular DDB2 molecules (85%) localize to the UV-damaged chromatin, only 25% of the XPC molecules are immobilized to the damaged DNA (Luijsterburg et al., 2007). Thus, it is important to examine different techniques to probe recruitment and departure of NER proteins. Interestingly, although simple immunoblotting of chromatin extract of post-irradiated samples did not reveal changes in accumulation kinetics of XPC, it revealed a clear shift in XPC mobility (Figure 5B), indicating a post-translational modification of XPC at the lesion site, which has often been suggested to be ubiquitination (Sugasawa et al., 2005; Wang et al., 2005). Thus, although inconclusive, such initial results from Western blot of sub-cellular fraction can provide a lead to the post-translational modification of XPC and similar proteins. This can then be further characterized by immunoprecipitation and mass spectrometry to identify specific sites of modification and study their impact on the NER functions of the protein.

#### Implication of Post-Translational Modifications in Recruitment and Departure of NER Proteins in NER

**FIGURE 6:**
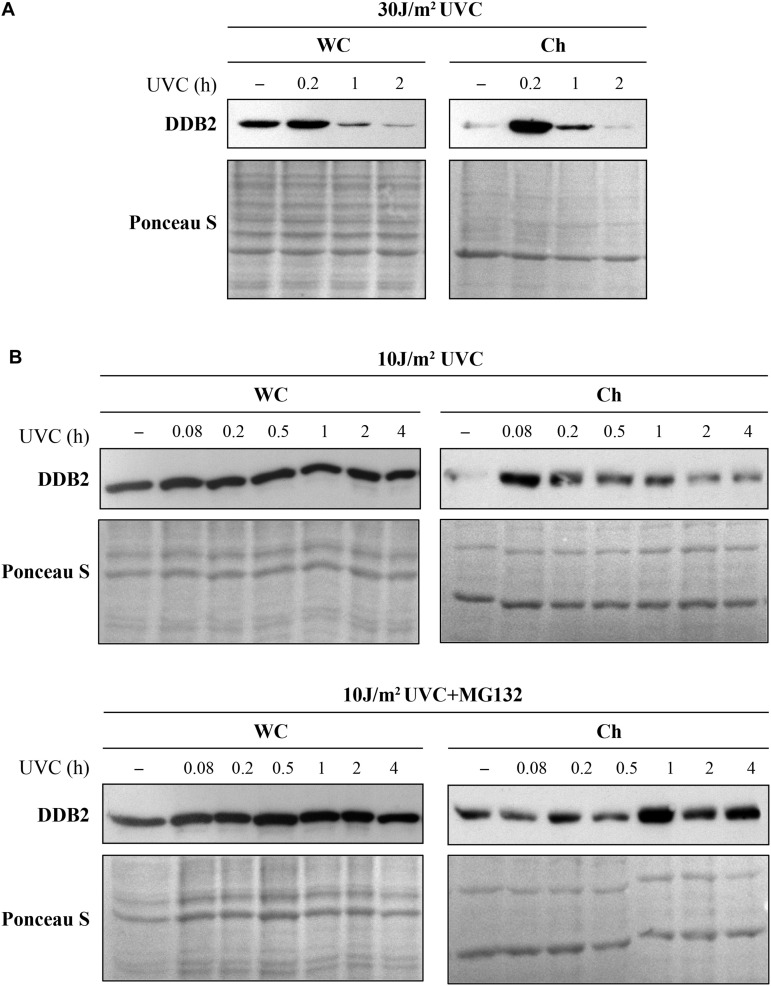
Ultraviolet radiation irradiation induces the DDB2 ubiquitination and degradation. The GMU6 cells were globally irradiated with UVC at 30 **(A)** or 10 J/m^2^ **(B)**, and fractionated at the indicated time. The whole cell extracts and chromatin-bound protein fractions were separated on SDS-PAGE and immunoblotted for DDB2. Where specified in panel **(B)**, 10 μM of the proteasome inhibitor MG132 was added 1 h before irradiation with 10 J/m^2^ UVC to show that the time-dependent depletion of DDB2 in Ch fraction is a result of proteasomal degradation of PTM-modified DDB2. Ponceau S staining was used as loading control.

Different PTM regulate the arrival and/or departure of repair proteins during NER (Dantuma and Van Attikum, 2016). Coupling cell fractionation with Western blot analysis offers complementary information on how the PTM affect the protein functions, whereas this information is not available if one uses total cell extracts for immunoblot analyses. Using proteasome and protein synthesis inhibitors in time course studies or modifying the acceptor sites to prevent a specific PTM, one can shed light on the role of a specific PTM in the repair process. For example, it is known that UVC (10 J/m^2^) induced binding of DDB2 to DNA lesions results in its auto-ubiquitination and proteasomal degradation by 3-6 h, followed by a complete recovery in 24 h (Rapic-Otrin et al., 2002; Puumalainen et al., 2014; Matsumoto et al., 2015). However, the extent of DDB2 degradation depends on the dose of UV. In cells irradiated with 30 J/m^2^ UVC, we noted reduction in DDB2 levels from 1 h after irradiation in both, whole cell extract and chromatin bound fraction of GMU6 cells (Figure 6A). However, in the cells exposed to lower doses of UVC (10 J/m^2^), the reduction in DDB2 levels from 0.2 to 4 h was visible in the chromatin-bound protein fractions but not in the whole cell extracts (Figure 6B, top panel) indicating that the DDB2 reduction is closely associated with its interaction with damaged DNA (Rapic-Otrin et al., 2002). To determine whether the reduction in DDB2 level at chromatin is due to just its departure from the lesion site or if there is a concomitant proteasomal degradation of the protein, we added the proteasome inhibitor MG132 to the medium 1 h before irradiation with UVC (10 J/m^2^). We noted that addition of proteasome inhibitor made no difference to the levels of DDB2 in the whole cell extracts up to 4 h, but suppressed its departure from the lesion site in the chromatin-bound protein fraction (Figure 6B, bottom panel). These results indicate that the reduction in the DDB2 signal at chromatin from 1 to 4 h is caused by proteasome-mediated DDB2 degradation, which confirms similar conclusion drawn in the previous studies (Rapic-Otrin et al., 2002; El-Mahdy et al., 2006).

### Combination of Above Protocols to Study the Influence of PARP1 on Function of XPA in NER

#### Influence of PARP1 on the kinetics of recruitment of XPA by improved local irradiation technique

**FIGURE 7:**
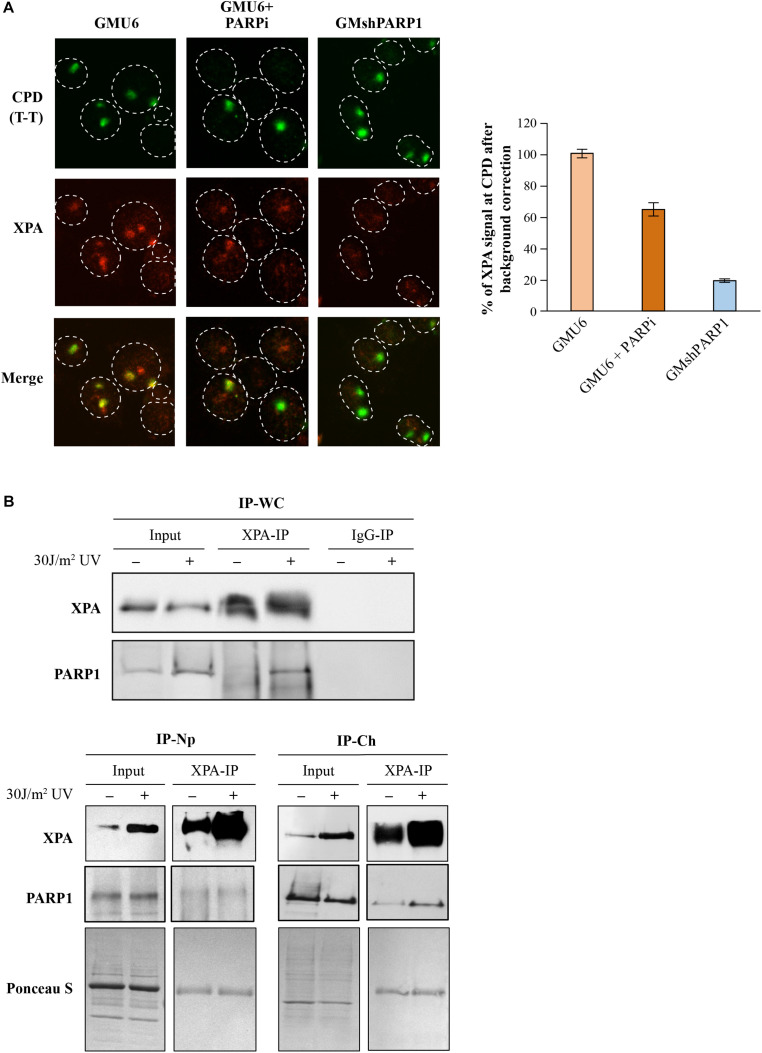
Influence of PARP1 and its catalytic activity on XPA functions. **(A)** PARP1 deficiency impairs XPA recruitment kinetics at the UVC-damaged chromatin. The PARP1-replete (GMU6), PARP1 depleted (GMshPARP1) and PARP-inhibitor treated PARP1-replete cells (PARPi) were locally irradiated with 100 J/m^2^ and processed after 30 min with C+T protocol. The XPA signal at T-T spots was quantified and corrected by subtracting the background nuclear signal for XPA measured in equivalent zones outside the local irradiation subnuclear spots. The data were pooled from at least 100 T-T spots derived from multiple slides from 3 independent experiments. **(B)** XPA and PARP1 interact with each other at the chromatin after UV irradiation. The GMU6 cells were globally irradiated with 30 J/m^2^ and fractionated after 10 min to obtain the Np and Ch protein fractions. The XPA- and IgG-IP (mock IP) were performed for the control and UV-treated fractions, followed by the detection of PARP1 and XPA. Ponceau S staining was used as loading control.

XPA was the first NER protein shown to interact with PAR (Pleschke et al., 2000) and the biochemical studies revealed the presence of a PAR specific binding domain in its C-terminal region (Fischer et al., 2014). To test the influence of the PARP1 and PAR on XPA kinetics, GMU6 cells were locally irradiated with UVC 30 min after treatment with PARP inhibitor, PJ-34, followed by the *in situ* extraction and immunofluorescence labeling. The accumulation of XPA at the local irradiation site was significantly reduced in the presence of PARP inhibitor and abrogated when PARP1 was depleted by shRNA, demonstrating that the recruitment of XPA to DNA lesion in NER is dependent on PARP1 and its catalytic activity (Figure 7A and chart).

#### Sub-cellular Fractionation as a Method to Detect UVC-induced Interaction of XPA with PARP1 in Chromatin-bound Fraction

The pulldown of a known NER protein followed by mass spectrometry identification of the interactome of this protein is the most exploited technique for identification of new players in NER. Many repair proteins form complexes with other proteins at the lesion site after induction of DNA damage (Boeing et al., 2016; Zhen and Yu, 2018), but some of them also interact with other proteins in the absence of any DNA damage (Isabelle et al., 2010). Therefore, immunoblotting and proteomic analyses of co-IP’s protein partners in each of the sub-cellular fractions (and not in the total cellular extracts) can provide information about the protein-protein interactions, such as when and where these interactions occur inside the cell, whether they do so before or after irradiation, whether they occur at the lesion site on the chromatin or outside in the nucleoplasm.

To exemplify, we examined the mode of interaction of XPA with PARP1 in different sub-nuclear compartments of GM skin fibroblasts before and after DNA damage (Figure 7B). The XPA-IP of the whole cell extracts revealed a significant UV-induced increase in the interaction between XPA and PARP1 (Figure 7B, top panel), which has also been reported earlier (King et al., 2012). PARP1 is a nuclear protein, therefore to identify the sub-nuclear compartment in which this complex was formed, we performed IP of nucleoplasmic and chromatin bound fraction. The IP of nucleoplasmic fraction of control and UV-irradiated GMU6 cells with XPA did not pull down PARP1, whereas the same IP performed in chromatin fraction revealed the UV-induced association of XPA with PARP1 (Figure 7B, bottom panels). Thus, the interaction of XPA with PARP1 in the whole cell extracts takes place only at the chromatin level. This limited interaction of PARP1 with XPA is in contrast with our previously reported interaction between PARP1 and XPC, which is not only at the chromatin after irradiation, but also before irradiation in both the nucleoplasm and chromatin extracts (Robu et al., 2017). In summary, the study of interaction of an NER protein with other protein partners, when carried out in the appropriate sub-cellular or sub-nuclear fraction would be far more informative in deciphering the role of their interactions on each other’s function in NER.

The repair of damaged DNA is crucial for maintaining genome integrity. A plethora of proteins are recruited to the lesion site in an orchestrated fashion to detect and remove the damage from chromatin. Although we have a good knowledge of minimal set of protein required for GGR, it is not yet clear how the NER recognition proteins can find the lesions so quickly in the compact structure of chromatin (Kusakabe et al., 2019). Moreover, it is becoming increasingly evident from the current research in the field of DNA repair that “accessory” proteins, i.e., those not involved in carrying out the core biochemical reactions of the repair, play multiples roles at the damage site, including modification of the chromatin environment to make it more accessible to the core repair proteins (Puumalainen et al., 2016; Gsell et al., 2020). Thus, the local UVC irradiation and in situ extraction coupled with indirect immunofluorescence and sub-cellular fractionation techniques allow us to gain more insights into the factors influencing the trafficking of NER proteins to and from damage site, identification of new factors and/or PTM involved in NER and understanding of the molecular mechanisms of this repair pathway. The protocols described here are centered on NER pathway, but they can be suitably modified to study a variety of biological processes, ranging from other DNA repair pathways to cell cycle and cell death.

## Data Availability Statement

The original contributions presented in the study are included in the article/supplementary material, further inquiries can be directed to the corresponding author.

## Author Contributions

MR and RS performed the experiments and analyzed the data. All authors conceived and designed the project, wrote and revised the manuscript, and read and approved the submitted version.

## Conflict of Interest

The authors declare that the research was conducted in the absence of any commercial or financial relationships that could be construed as a potential conflict of interest. The handling editor declared a shared affiliation with the authors at the time of review.
